# The amino acid transporter AAP1 mediates growth and grain yield by regulating neutral amino acid uptake and reallocation in *Oryza sativa*

**DOI:** 10.1093/jxb/eraa256

**Published:** 2020-06-02

**Authors:** Yuanyuan Ji, Weiting Huang, Bowen Wu, Zhongming Fang, Xuelu Wang

**Affiliations:** 1 State Key Laboratory of Genetic Engineering, Department of Genetics, School of Life Sciences, Fudan University, Shanghai, China; 2 Key Laboratory of Plant Resource Conservation and Germplasm Innovation in Mountainous Region (Ministry of Education), College of Agricultural Sciences, Guizhou University, Guiyang, China; 3 National Key Laboratory of Crop Genetic Improvement, Center of Integrative Biology, College of Life Science and Technology, Huazhong Agricultural University, Wuhan, China; 4 University of Birmingham, UK

**Keywords:** Amino acid, grain yield, nitrogen, transporter, OsAAP1, rice, tillering

## Abstract

Nitrogen (N) is a major element necessary for crop yield. In most plants, organic N is primarily transported in the form of amino acids. Here, we show that amino acid permease 1 (AAP1) functions as a positive regulator of growth and grain yield in rice. We found that the *OsAAP1* gene is highly expressed in rice axillary buds, leaves, and young panicles, and that the OsAAP1 protein is localized to both the plasma membrane and the nuclear membrane. Compared with the wild-type ZH11, *OsAAP1* overexpression (OE) lines exhibited increased filled grain numbers as a result of enhanced tillering, while RNAi and CRISPR (clustered regularly interspaced short palindromic repeat; *Osaap1*) knockout lines showed the opposite phenotype. In addition, *OsAAP1*-OE lines had higher concentrations of neutral and acidic amino acids, but lower concentrations of basic amino acids in the straw. An exogenous treatment with neutral amino acids promoted axillary bud outgrowth more strongly in the OE lines than in the WT, RNAi, or *Osaap1* lines. Transcriptome analysis of *Osaap1* further demonstrated that OsAAP1 may affect N transport and metabolism, and auxin, cytokinin, and strigolactone signaling in regulating rice tillering. Taken together, these results support that increasing neutral amino acid uptake and reallocation via OsAAP1 could improve growth and grain yield in rice.

## Teaser

OsAAP1 positively regulates growth and grain yield in rice by influencing amino acid uptake and reallocation.

## Introduction

Nitrogen (N) is one of the major nutrients for plant growth and crop yield. Most non-legume plants absorb inorganic N, including nitrate and ammonium, which are then utilized in amino acid synthesis. These amino acids, which are the primary forms of transported organic N, are then transported and reallocated from the source organs (e.g. developing roots and leaves) to the sinks (such as flowers, fruits, and seeds) through the xylem and phloem (Lam *et al.*, 1996; Xu *et al.*, 2012). In addition, plants also directly take up amino acids from soil (Nasholm and Persson, 2001; Tegeder and Masclaux-Daubresse, 2018). Recent studies have shown that an increased loading of amino acids in phloem and embryos may improve the biomass and seed yield of pea (Zhang *et al.*, 2015; Perchlik and Tegeder, 2017).

Plants require the membrane-integral amino acid transporter proteins (AATs) to absorb and translocate amino acids. A large number of *AAT* genes have been identified in Arabidopsis (Tegeder and Ward, 2012), rice (Zhao *et al.*, 2012), potato (Ma *et al.*, 2016), and poplar (Wu *et al.*, 2015). Among them, the amino acid permease (AAP) subfamily, which function as proton-coupled amino acid transporters with broad substrate specificity, have been intensively studied

(Fischer *et al.* 2002; Tegeder and Masclaux-Daubresse, 2018). In Arabidopsis, *AtAAP1* is involved in both root uptake and embryo loading of neutral amino acids, both of which affect the seed yield (Lee *et al.*, 2007; Sanders *et al.*, 2009; Perchlik *et al.*, 2014). *AtAAP8* is expressed in the source leaf phloem and functions in phloem amino acid loading, and knockout of *AtAAP8* resulted in decreased silique and seed numbers. Similarly, *StAAP1* functions in the long-distance transport of amino acids from source leaves to sink tubers in potato (Koch *et al.*, 2003). PtAAP11 has a high affinity for proline and may function in xylogenesis in poplar (Couturier *et al.*, 2010). In rice, through a genome-wide survey of *AAT* genes, 19 *AAP* genes (*OsAAP1–OsAAP19*) were identified (Zhao *et al.*, 2012). Among them, *OsAAP6* was cloned by quantitative trait locus mapping and has been demonstrated to enhance grain protein content by regulating the synthesis and accumulation of grain storage proteins (Peng *et al.*, 2014). OsAAP8 participates in the transport of amino acids to developing grains and increases grain yield by remobilization of N to grains (Pereira *et al.*, 2015). OsAAP1, OsAAP3, OsAAP7, and OsAAP16 have been examined for their amino acid transport ability in *Xenopus laevis* oocytes using electrophysiology. Like AtAAP1 in Arabidopsis, OsAAP1, OsAAP7, and OsAAP16 transport neutral amino acids, though their biological function in rice is unclear (Taylor *et al.*, 2015). In contrast, OsAAP3 had a distinct substrate specificity for basic amino acids, including Lys and Arg (Taylor *et al.*, 2015; Lu *et al.*, 2018). OsAAP5 has been shown to have similar substrates to OsAAP3 (Wang *et al.*, 2019).

Tillering/shoot branching is one of the most important agronomic traits that determines rice grain yield. Tiller number is regulated by many hormones and external environmental factors. The phytohormones auxin and strigolactone (SL) inhibit shoot branching in Arabidopsis (Leyser, 2003; Gomez-Roldan *et al.*, 2008; Umehara *et al.*, 2008; Wang *et al.*, 2013; Hu *et al.*, 2019), while cytokinin promotes shoot branching in Arabidopsis (Dun *et al.*, 2012), and brassinosteroids promotes tillering in rice (Fang *et al.*, 2020). In addition, the ammonia N content in soil is positively correlated with rice tiller number (Sasaki *et al.*, 2002). An elevated NH_4_NO_3_ level, commonly used as a high-N fertilizer, could also promote the tiller number of rice (Li *et al.*, 2016). In support of the important role for N in crop yield, the nitrate transporter 1/peptide transporter family (NPF) gene members *OsNPF7.1*, *OsNPF7.2*, *OsNPF7.3*, *OsNPF7.7*, and *OsNPF8.20* positively regulate tiller number in rice, whereas *OsNPF7.4* plays a negative role in regulating rice tillering (Fang *et al.*, 2013, 2017; Huang *et al.*, 2018, 2019; Wang *et al.*, 2018). Moreover, the basic amino acid transporters OsAAP3 and OsAAP5 function as negative regulators in rice tillering and grain yield (Lu *et al.*, 2018; Wang *et al.*, 2019).

In this study, we found that *OsAAP1* was highly expressed in the roots, axillary buds, leaves, and young panicles. OsAAP1–green fluorescent protein (GFP) is localized on both the plasma membrane and nuclear membrane of rice protoplasts. Overexpression of *OsAAP1* led to an increased number of filled grains per plant as a result of an increased tiller number in rice. Knockout of *OsAAP1* affected N transport and metabolism, and many hormonal pathways, resulting in the inhibition of axillary bud outgrowth and reduced tiller number. Thus, our results provide a valuable candidate gene for the improvement of rice yield.

## Materials and methods

### Construction of *OsAAP1* lines with altered expression and promoter–GUS (β–glucuronidase) transgenic lines

To construct the *OsAAP1* overexpression (OE) vector, a 1464 bp *OsAAP1* (LOC_Os07g04180) cDNA was amplified using the primers 5'-GGGGTACCATGGGGATGGAGAGGCCGCAA-3' and 5'-GCTCTAGATGAGGAGACGCTGAATGGCTT-3′, and inserted downstream of the *35S* promoter in pCAMBIA1306 [a binary vector modified by adding the 35S promoter, FLAG tag, and terminator to the backbone of pCAMBIA1300 (Roberts *et al.*, 1996)] using *Kpn*I and *Xba*I restriction enzymes (Wang *et al.*, 2017). To generate the *OsAAP1*-RNAi vector, two fragments of *OsAAP1* cDNA (217 bp) were amplified by PCR using the primers 5'-AGGATCCTGGGGATGGAGAGGCCGCAA-3′ and 5'-AAGGTACCATCACCCACCCCAGCTGCGCTA-3′; 5'-AGAGCTCTGGGGATGGAGAGGCCGCAA-3′; and 5'-AAACTAGTATCACCCACCCCAGCTGCGCTA-3′, and transferred downstream of the *Ubi-1* promoter in the rice RNAi vector pTCK303 (Wang *et al.*, 2004) using *Bam*HI/*Kpn*I and *Spe*I/*Sac*I, respectively. For the *pOsAAP1::GUS* vector, a 2500 bp fragment of the *OsAAP1* promoter (upstream of the start codon ATG) was cloned using the primers 5'-ACCATGATTACGCCAAGCTTGCTGAAGAGAAGACTCGAGTGTTG-3′ and 5'-CAGTGAATTCCCGGGGATCCTTCAATTCCTAGCTTAGCTAGCGG-3′, and inserted up to the pCAMBIA1391Z vector with *Hin*dIII and *Bam*HI (Roberts *et al.*, 1996).

All of the aforementioned vectors were introduced into the *Agrobacterium tumefaciens* strain *EHA105*, and Zhonghua11 (ZH11) was transformed with these vectors as the background.

The T_2_ homozygous transgenic lines (OE and RNAi) with altered expression were selected using quantitative real-time PCR (qRT-PCR). The T_1_ transgenic lines of the *OsAAP1* promoter GUS line were used for GUS staining.

### GUS signal analysis of the *OsAAP1* promoter

Histochemical GUS assays were processed as described previously (Sieburth and Meyerowitz, 1997). First, different tissues collected from *OsAAP1* promoter GUS transgenic lines were vacuum infiltrated for 15 min, and fixed in FAA (formalin:acetic acid:70% ethanol 1:1:18 v/v/v) at 4 °C for 20–30 min. The samples were then incubated in staining buffer at 37 °C overnight. After discoloring by incubation in a solution of 75% ethanol, the stained samples were observed using a stereo microscope. To make sections, the stained tissues were rinsed and fixed in FAA at 4 °C for 24 h, gradually dehydrated with ethanol for 15 min each time, and washed twice with 100% ethanol for 30 min each time. Finally, the samples were embedded in Spurr resin, and ultramicrotome sections (2–8 μm) were applied onto poly-l-lysine-coated slides with glass knives. The sections were observed using Zeiss Axio Imager M2 (Carl Zeiss AG, Oberkochen, Germany).

### Quantitative RT-PCR analysis

Total RNA was extracted using Trizol reagent according to the manufacturer’s instructions (Invitrogen). The cDNA was synthesized from 3 μg of total RNA from each sample using M-MLV reverse transcriptase (Promega). qRT-PCR was performed to monitor gene expression, carried out with SYBR Green (Takara), and monitored in real-time with a 7500 qRT-PCR system (Applied Biosystems, USA). The primers for gene expression analysis of *OsAAP1* were 5'-GCACATTACAAGCCATTCAGCGTC-3′ and 5'-CTGACGAAACACTTGAGCACTC-3′. *OsACTIN1* (LOC_Os03g50885) was used as an internal standard with the primers 5'-CGGTGTCATGGTCGGAAT-3′ and 5'-GCTCGTTGTAGAAGGTGT-3′.

### Protoplast preparation

Our protoplast isolation protocol was based on the protocol for Arabidopsis protoplasts provided online by J. Sheen’s laboratory 9http://genetics.mgh.harvard.edu/sheenweb/0 with several modifications. At 7 d to 14 d post-germination, rice plants were ~10–20 cm tall. The plant materials were put in balance liquid (0.6 M mannitol), and then shoot tissue was cut into 0.5 mm pieces using very sharp razors and placed into the balance liquid for 10 min. Tissue was immediately incubated in an enzyme solution [10 ml containing 0.6 M mannitol, 10 mM MES (pH 5.7), 1.5% Cellulase RS, 0.75% Macerozyme, 0.1% pectinase, 0.1% BSA, and 1 mM CaC1_2_] for 4 h in the dark under gentle shaking (80 rpm). After incubation, protoplasts were passed through a Centauri. A 10 ml aliquot of W5 solution [154 mM NaCl, 125 mM CaC1_2_, 5 mM KC1, 2 mM MES (pH 5.7)] was added and the solution was centrifuged for 5 min at 80 *g* to pellet the protoplasts.

### Transient expression of *OsAAP1* in rice protoplasts and *Nicotiana benthamiana* leaves

To construct the *OsAAP1* subcellular localization vector, the primer sequences 5'- CTGTACAAGAGCGGCCGCATGGGGATGGAGAGGCCGCAAG-3′ and 5'-CAAATGTTTGAACGATCTGCAGTGAGGAGACGCTGAATGGCTTGT-3′ were used to amplify *OsAAP1* cDNA, which was then inserted downstream of the *35S* promoter of the HBT-GFP-NOS vector using *Not*I and *Pst*I to produce the *GFP*-*OsAAP1* vector with the stop codon of *GFP* removed (Sheen, 1993). *OsAAP1* cDNA was also amplified using the primers 5'-CGGTACCCGGGGATCCTCTAGAATGGGGATGGAGAGGCCGCAA-3′ and 5'-GATTCGTTCTTTACTGTCGACTGAGGAGACGCTGAATGGCTTGT-3′, and inserted downstream of the *35S* promoter of pCAMBIA2302 vector [a binary vector modified by adding the 35S promoter, GFP tag, and terminator to the backbone of pCAMBIA2300 (Roberts *et al.* 1996)] to generate the *2302-OsAAP1-GFP* construct (Cheng *et al.*, 2014). Rice protoplasts were re-suspended in Mmg solution [0.6 M mannitol, 15 mM MgC1_2_, 4 mM MES (pH 5.7)], and 100 μl of rice protoplast suspension (~5×10^5^ cells) was transfected with 60 μg of *HBT* and *OsAAP1-GFP* with the polyethylene glycol (PEG) transformation method (Yoo *et al.*, 2007), followed by incubating in W5 medium at 28 °C for 16 h before confocal (TCS SP8; Leica) examination. The *Agrobacterium* strain *GV3101* transformed with 2302-*OsAAP1* was infiltrated into 1-month-old *N. benthamiana* plants to study the transient expression of *OsAAP1*. The DAPI, GFP, and FM4-64 fluorescence signals were detected at 2 d post-injection using the confocal laser scanning microscope.

### Hydroponic culture and field experiments

Hydroponic experiments were conducted using a basic rice culture solution (Yoshida *et al.*, 1976) under natural rice growth conditions (the greenhouse condition is 32 °C in the daytime and 25 °C in the evening with a 400 W sodium lamp). N content and N source were adjusted in each experiment. Tiller number and effective panicle number were counted at the filling stage. Field experiments were carried out in an experimental field at Huazhong Agricultural University, China, during the rice growing season in 2016–2018.

### Amino acid, soluble protein, and total nitrogen analysis

Total free amino acid and total soluble protein contents were measured by the ninhydrin method and the Bradford method, respectively, as described in Fang *et al.* (2013). Single free amino acid concentrations were measured using HPLC with an amino acid analyzer (L-8800 Hitachi). The samples were prepared as follows: 1 g of rice tissue (root, leaf sheath, or leaf) was placed in 80% ethanol (10 ml) at 80 °C in a water bath for 20 min and the supernatant was collected; this step was repeated twice. The collected extracts were placed at 80 °C in a drying oven to remove the ethanol and water, and 1 ml of 0.5 M NaOH was used to dissolve the sediment. The solution was centrifuged at 16 837 *g* for 15 min. The supernatant was collected and filtered through a filter membrane (2 μm); 0.8 ml of each filtrate was analyzed using the amino acid analyzer. Total N content was determined using the semi-micro Kjeldahl method using an N analyzer (Smart Chem 200, Westco, Italy) (Cavett, 1931). Nitrogen utilization efficiency (NUtE) was determined by the formula:

$$\begin{array}{ll} NUtE\left(\%\right)= & grain\;yield\;\left(g\right)/grain\;nitrogen\;content\;\left(g\right)\\ + & straw\;nitrogen\;content\;\left(g\right)] \end{array}$$

### Protoplast amino acid uptake assay

Amino acids with fluorescein isothiocyanate (FITC) markers (Pro–FITC, Ala–FITC, and Tyr–FITC) were synthesized by Yuan Peptide Biotechnology Company. A protoplast amino acid uptake assay was performed as described previously (Rottmann *et al.*, 2018). Rice protoplasts prepared from the transgenic lines and the wild-type ZH11 were kept in 1 ml of W5 buffer (154 mM NaCl, 125 mM CaCl_2_, 5 mM KCl, and 2 mM MES, pH 5.8) containing each FITC-labeled amino acid to a final concentration of 1 mM in the dark at 28 °C for 4 h, the protoplasts were centrifuged at 100 *g* for 5 min and the supernatant was carefully removed, and then the protoplasts were suspended with 1 ml of W5 solution and centrifuged at 100 *g* for 5 min. This step was repeated three times. Finally, the fluorescence signals were observed using a confocal laser scanning microscope.

### Nitrogen treatment assays

To detect whether the *OsAAP1* expression responded to various N supplies, 3-week-old *pOsAAP1::GUS* lines were transferred to the N-free basic nutrient solution for 3 d, and then transferred to N-free basic nutrient solution with or without 2 mM Pro, Ala, Tyr, Arg, Lys, Asp, Glu, NaNO_3_, (NH_4_)_2_SO_4_, and NH_4_NO_3_ for 4 h, respectively. To investigate the effect of neutral amino acids on the *OsAAP1* transgenic plants, seedlings were grown in basic rice culture solution with 1 mM NH_4_NO_3_ as the N source for 1 week, then transferred to the basic rice culture solution supplemented with 0.5 mM neutral amino acids (mixed with an equal amount of Ser, Gly, Ala, Cys, Val, Met, Ile, Leu, Tyr, Phe, and Pro) as the N source for an additional 3 weeks. To further explore the effect of different amino acid groups on the *OsAAP1* transgenic plants, seedlings were grown in basic rice culture solution for 1 week, then transferred to basic rice culture solution with 1 mM basic amino acids Lys and Arg, 1 mM acidic amino acids Asp and Glu, or 1 mM neutral amino acids Tyr and Pro for an additional 3 weeks, respectively. To analyze the phenotype of *OsAAP1* transgenic plants in the presence of different concentrations of NH_4_NO_3_, 1-week-old seedlings were cultivated in basic rice culture solution supplemented with 0.25, 1.0, or 4.0 mM NH_4_NO_3_ for an additional 3 weeks. The nutrient solution was renewed every 3 d.

### Construction of *OsAAP1* CRISPR lines

The *OsAAP1* CRISPR (clustered regularly interspaced short palindromic repeat) vector construct was prepared using CRISPR/CRISPR-associated protein 9 (Cas9)-based multiplex genome editing for monocot and dicot plants (Ma *et al.*, 2015). Two target sequences (5'-TGGCGTTCTCGGTCATAACCTGG-3′ and 5'-CCAAGCCGTCAGGGCCAACCTAG-3′) of *OsAAP1* were designed and added to the U6IPAT/U6OPST or the U3IPAT/U3IPST primers. A 422 bp fragment from target sequence 1 was amplified by PCR using primers U6OPST and *OsAAP1*-U6IPAT (5'-GGTTATGACCGAGAACGCCACAACCTGAGCCTCAGCGCAGC-3′) and the U6 plasmid. In the same way, a 348 bp fragment was amplified by PCR from target sequence 2 using primers ZRO589 and *OsAAP1*-U3IPAT (5'-GGTTATGACCGAGAACGCCATGCCACGGATCATCTGCACAACTC-3′) with the U3 plasmid. A 476 bp fragment from target sequence 1 was amplified by PCR using primers *OsAAP1*-U6OPST (5'-GTGGCGTTCTCGGTCATAACCGTTTTAGAGCTAGAAATAGCAAGTTA-3′) and U6IPAT with the U6 plasmid. Similarly, a 463 bp fragment from target sequence 2 was amplified by PCR using primers *OsAAP1*-U3IPST (5'-ATGGCGTTCTCGGTCATAACCGTTTTAGAGCTAGAAATAGCAAGTTA-3′) and ZRO282 with the U3 plasmid. Complete U6 and U3 fragments were amplified by fusion PCR containing U6 promoter–short guide RNA (sgRNA) and U3 promoter–sgRNA. The U6 fragment was digested with *Kpn*I, and the U3 fragment was digested with *Kpn*I and *Sac*I, and then the U6 and U3 fragments were connected. The complete U6 and U3 sequence of 1600 bp was amplified by PCR using U6OPST and ZRO282 primers with the U6–U3 plasmid. Finally, the entire U6–U3 fragment of 1600 bp was inserted into the Per8–Cas9 vector using *Kpn*I and *Sac*I, and cloning kits. The vector was transformed into the ZH11 background. The T_0_ CRISPR transgenic lines were selected by PCR and sequencing using the corresponding primers 5'-GTCCGGTGTCCATGGATGAGGTAG-3′ and 5'-CACCTCAGAAGGTGTACACAGTC-3′.

### RNA-seq analysis

The axillary buds (<0.5 mm) from *OsAAP1-CRISPR* lines and the wild-type ZH11 plants were collected for RNA sequencing (RNA-seq), analysis and two biological replicates were performed for each sample by Novogene. The clean data were aligned to the rice genome reference sequence (*Oryza_sativa*.IRGSP-1.0) by HiSAT2 (v2.1.0) (Kim *et al.*, 2015). Transcripts were then assembled by stringtie (v2.0.1) (Pertea *et al.*, 2016) and then processed by featureCounts to summarize the counting reads (subread-2.0.0) (Liao *et al.*, 2014). The intersection of differential genes analyzed by DESeq2 [false discovery rate (FDR) <0.05 and fold change ≥2] were identified as differentially expressed genes (DEGs) (Love *et al.*, 2014).

### Statistical analysis

Differences were analyzed using Student’s *t*-test. Significance levels: ***P < 0.001; **P < 0.01; *P < 0.05.

## Results

### OsAAP1 is highly expressed in rice axillary buds, leaves, and young panicles

**Fig. 1.**
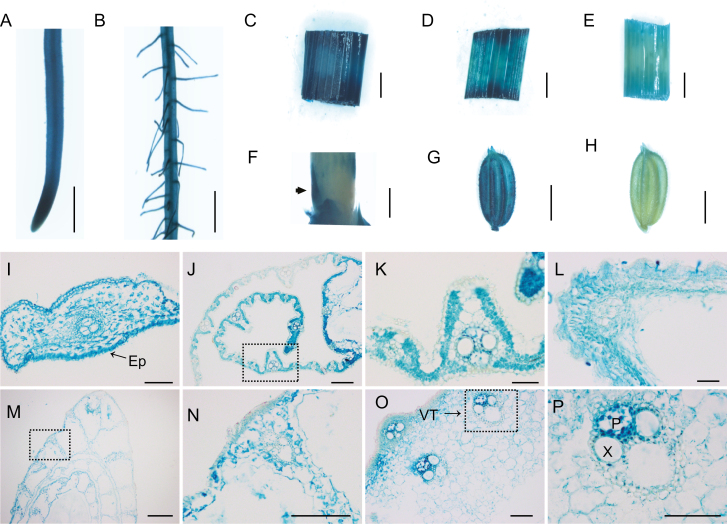
The *pOsAAP1*::GUS staining in the root tip (A), lateral root (B), leaf sheath (C), leaf blade (D), culm (E), tiller base (F), and panicle (G and H). Transverse section of the root tip (I), leaf blade (J and K), the young panicle (L), leaf sheath (M and N), and culm (O and P) from the *pOsAAP1*::GUS transgenic plants. Ep, epidermis; VT, vascular tissue; P, phloem; X, xylem. The arrow indicates the axillary bud in (F). The dotted frame in (J), (M), and (O) indicates the region enlarged in (K), (N), and (P), respectively. Scale bars represent 5 mm (A–H) and 200 μm (I–P).

To elucidate the biological functions of *OsAAP1*, we first generated *pOsAAP1::GUS* transgenic plants in the ZH11 background; the GUS staining results showed that *OsAAP1* was highly expressed in root tips (Fig. 1A), lateral roots (Fig. 1B), leaf sheaths (Fig. 1C), leaf blades (Fig. 1D), culm (Fig. 1E), axillary buds (Fig. 1F), and young panicles (Fig. 1G), but was only slightly expressed in seeds at the filling stage (Fig. 1H). Furthermore, GUS activity was abundant in the transverse section of roots (Fig. 1I), and in the epidermis, cortex, and vascular bundles of the longitudinal section of leaf blades (Fig. 1J, K). Moreover, the GUS signal was widely distributed in the transverse sections of young panicles (Fig. 1L) and leaf sheaths (Fig. 1M, N). In the culm, the GUS signal was detected in parenchyma cells of the cortex and vascular tissues, and was stronger in companion cells of the phloem (Fig. 1O, P). The root of the wild-type ZH11 was stained as the negative control with no GUS signal detected (Supplementary Fig. S1 at *JXB* online). Then, we performed qRT-PCR using RNAs isolated from diverse tissues collected from the wild-type ZH11, including roots, leaf sheaths, axillary buds, tiller bases, and leaf blades at the vegetative stage, and stems, panicles, and leaf blades at the reproductive stage. These results showed that *OsAAP1* was widely expressed in many rice tissues, but was much more highly expressed in roots, mature leaf blades, and panicles (Supplementary Fig. S2A), indicating that *OsAAP1* may play a role in uptake of amino acids in the roots and loading of amino acids in the whole plant.

To detect the transcriptional response of *OsAAP1* to various N treatments, 3-week-old wild-type ZH11 seedlings were transferred to the N-free basic nutrient solution for 3 d and then transferred to various N source solutions for 4 h. GUS staining showed that the *OsAAP1* expression was decreased in root tips and lateral roots with 2 mM Pro, Ala, Tyr, Arg, Lys, or nitrate, but was increased with 2 mM Asp, Glu, ammonium, and ammonium nitrate (Supplementary Fig. S2B). These results indicated that *OsAAP1* expression was regulated by different N sources. In addition, we used hormones [cytokinin, auxin, SL, and abscisic acid (ABA)] to treat wild-type rice seedings to explore whether they could affect the expression of *OsAAP1*. Interestingly, we found that ABA and the SL analog *rac*-GR24 could significantly inhibit the *OsAAP1* expression, while indole-3-acetic acid (IAA) and 6-benzyl adenine (6-BA) did not affect its expression (Supplementary Fig. S2C). Moreover, an ABA-responsive element (ABRE; ACGTGGC) has been identified in a previous study (Mundy *et al.*, 1990; Choi *et al.*, 2000), We scanned the promoter sequence of *OsAAP1* (2500 bp upstream of ATG) and found two ACGT-containing elements (ACEs) and one ABRE, which may be responsible for ABA’s regulatioin of *OsAAP1* expression (Supplementary Fig. S2D).

### OsAAP1 localized both on the plasma membrane and the nuclear membrane

**Fig. 2.**
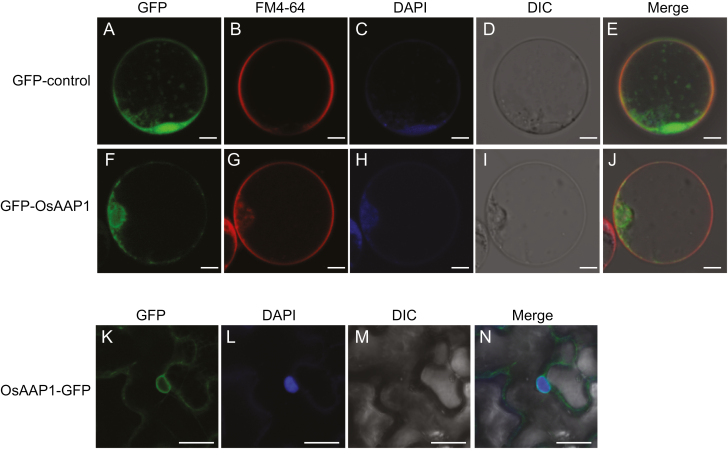
Subcellular localization of OsAAP1–GFP. (A–E) Localization of the 35S promoter-driven *GFP* as the control in rice protoplasts. (F–J) Localization of the 35S promoter-driven GFP–OsAAP1 in rice protoplasts. (K–N) Localization of the 35S promoter-driven OsAAP1–GFP in tobacco pavement cells. Green, GFP signal. Red, FM4-64 (a lipophilic membrane marker) signal. Blue, DAPI (a nuclear marker) signal. DIC, the bright field. Scale bars represent 5 μm in (A–J) and 25 μm (K–N).

OsAAP1 was predicted by the TMHMM Server (http://www.cbs.dtu.dk/services/TMHMM/) to have nine transmembrane helixes with the N-terminus in the cytoplasmic side. To investigate the subcellular localization of OsAAP1, a *35S* promoter-driven *GFP* fused to *OsAAP1* was transiently expressed in rice protoplasts. We found that the GFP signal was widely observed in rice protoplasts (Fig. 2A–E), but that GFP–OsAAP1 mainly localized on the nuclear membrane and plasma membrane (Fig. 2F–J). The plasma membrane was labeled by FM4-64 (Fig. 2B, G) (Shi *et al.*, 2012). The nuclear membrane localization was further confirmed by DAPI staining for DNA (Fig. 2C,H) (Sakamoto *et al.*, 2004). In addition, the *35S* promoter-driven *OsAAP1* with a *GFP* tag at the C-terminus (2302-*OsAAP1-GFP*) was transiently expressed in pavement cells of *N. benthamiana*. The GFP signal was detected not only on the plasma membrane but also in the endomembrane system in the cytosol. DAPI staining further confirmed that OsAAP1–GFP was also localized on the nuclear membrane (Fig. 2K–N). Together, these results indicated that OsAAP1 is localized on both the plasma membrane and the nuclear membrane, which is similar to the subcellular localization of AtAAP3 in Arabidopsis (Okumoto *et al.*, 2004).

### Overexpression of *OsAAP1* improved grain yield by increasing tiller number and filled grain number

**Fig. 3.**
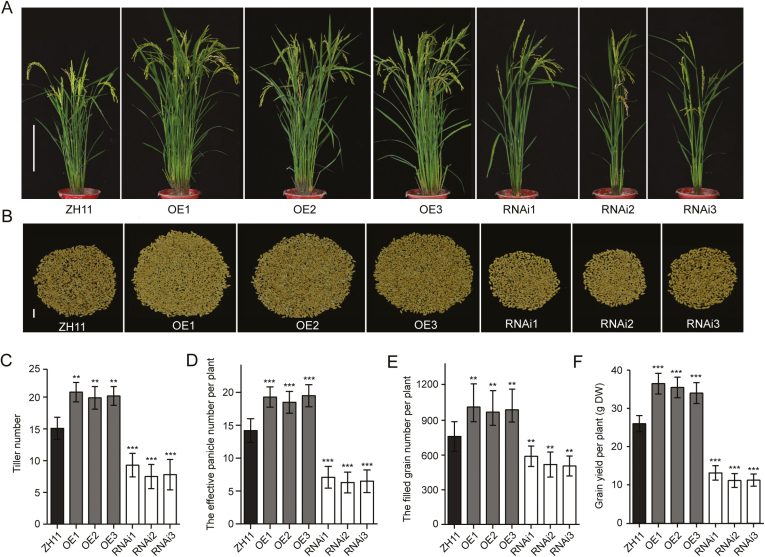
*OsAAP1* promotes tillering and biomass accumulation. (A) The phenotype of the whole plants of the *OsAAP1* OE lines, the RNAi lines, and the wild-type ZH11. (B) The filled grain number per plant shown in (A). (C) Quantification of tiller number of the plants shown in (A). (D) Quantification of the effective panicle number per plant shown in (A). (E) Quantification of the filled grain number per plant shown in (B). (F) Quantification of grain yield per plant (g DW) shown in (B). Scale bars represent 25 cm in (A) and 3 cm in (B). Dark gray, gray, and white bars represent ZH11, OE, and RNAi plants, respectively. OE1–OE3 indicate the different *OsAAP1*-overexpression lines, and RNAi1–RNAi3 indicate the different *OsAAP1-RNAi* lines. ZH11 indicates the wild type. Data are represented as the mean ±SD (*n*>20). Differences were analyzed using Student’s *t*-test. ***P<0.001, **P<0.01, *P<0.05.

To clarify the role of *OsAAP1* in rice growth and development, we constructed the OE and RNAi lines of *OsAAP1* in rice. The expression levels of *OsAAP1* in the three OE lines were significantly higher than in the wild-type ZH11, while the RNAi lines showed markedly reduced *OsAAP1* expression compared with the wild type (Supplementary Fig. S3). The OE lines showed increased tiller number and effective panicle number at the reproductive stage compared with the wild-type ZH11 (Fig. 3A, C, D) in the field. In addition, overexpression of *OsAAP1* resulted in enhanced filled grain number and grain yield per plant (Fig. 3B, E, F). Moreover, we found that *OsAAP1* OE lines significantly improved the total N content in straw (Supplementary Fig. S4A), grains per plant (Supplementary Fig. S4B), and the NUtE per plant as compared with the wild-type counterparts (Supplementary Fig. S4C), while *OsAAP1* RNAi lines showed the opposite phenotype (Fig. 3; Supplementary Fig. S4). Therefore, it was suggested that *OsAAP1* can promote tiller number and NUtE, leading to increased rice grain yield at the individual plant level.

### OsAAP1 may take up and transport neutral amino acids in rice

**Fig. 4.**
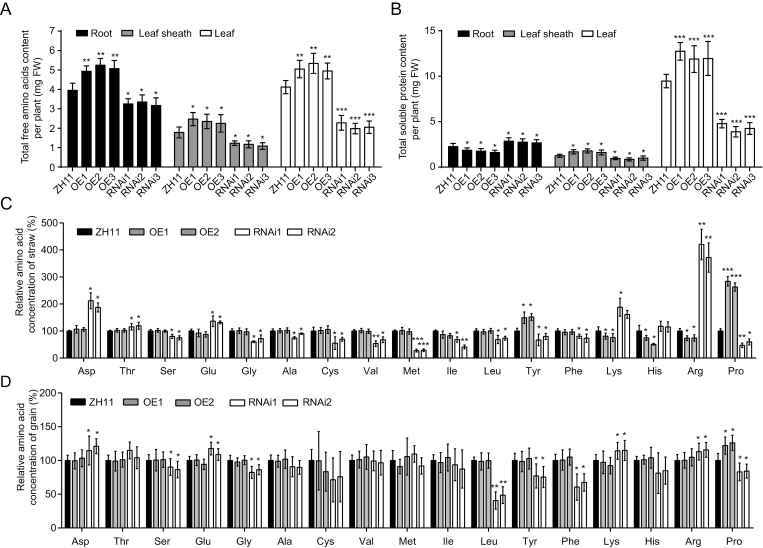
OsAAP1 promotes neutral amino acid transportation in rice. (A and B) Total free amino acids and soluble protein content per plant of the wild-type ZH11, the *OsAAP1* OE lines, and the RNAi lines. Dark gray, gray, and white bars represent root, leaf sheath, and leaf, respectively. (C and D) Amino acid concentrations in the straw and the grain relative to the wild-type ZH11 of the *OsAAP1*-OE lines and the RNAi lines, respectively. Dark gray, gray, and white bars represent ZH11, OE, and RNAi transgenic plants, respectively in (C and D). Data are represented as the mean ±SD. Differences were analyzed using Student’s *t*-test. ***P<0.001, **P<0.01, *P<0.05.

To investigate the effect of altered *OsAAP1* expression on amino acid and protein allocation, total free amino acid content (Fig. 4A) and total soluble protein content per plant (Fig. 4B) were measured in the root, leaf sheath, and leaf of the OE and RNAi transgenic lines under a low ammonium nitrate concentration (0.25 mM). Total free amino acids in the three tissues were greatly enhanced in the OE lines but significantly reduced in the RNAi lines compared with those in the wild-type ZH11 (Fig. 4A). However, the total soluble protein content was decreased in the roots of OE lines, but predominantly increased in the roots of RNAi lines (Fig. 4B). Furthermore, total soluble protein content per plant in the leaf sheath and leaf was notably increased in the OE lines but significantly decreased in the RNAi lines compared with the wild type (Fig. 4B). These results suggest that overexpression of *OsAAP1* may strengthen the transport of amino acids from the root to the aboveground parts and promote amino acid assimilation to soluble proteins in the aboveground parts, leading to the decreased amino acid levels in roots.

Furthermore, we measured the concentration of individual amino acids in the straw and grain of the *OsAAP1* transgenic lines. The results showed that most neutral amino acids, including Ser, Gly, Ala, Cys, Val, Met, Leu, Ile, Tyr, Phe, and Pro, were found in lower concentrations in the straw of the RNAi lines than in that of ZH11 (Fig. 4C; Supplementary Table S1). In addition, the concentrations of Tyr and Pro in the straw of the OE lines were predominantly increased as compared with those in the wild-type ZH11, but other neutral amino acid contents were unchanged (Fig. 4C; Supplementary Table S1). The contents of the basic amino acids Lys and Arg and the acidic amino acids Asp and Glu, however, were significantly increased in both the straw and grain of the RNAi lines (Fig 4C, D). In contrast, the content of Lys and Arg were lower in the straw of the OE lines compared with that of the wild-type ZH11 (Fig. 4C; Supplementary Table S1). Moreover, the concentrations of neutral amino acids, including Ser, Gly, Leu, Tyr, Phe, and Pro, were significantly decreased in the grains of the RNAi lines, while only the Pro content was slightly increased in the grains of the OE lines compared with those of the wild-type ZH11, indicating that overexpression did not affect the seed quality of rice (Fig. 4D; Supplementary Table S2).

**Fig. 5.**
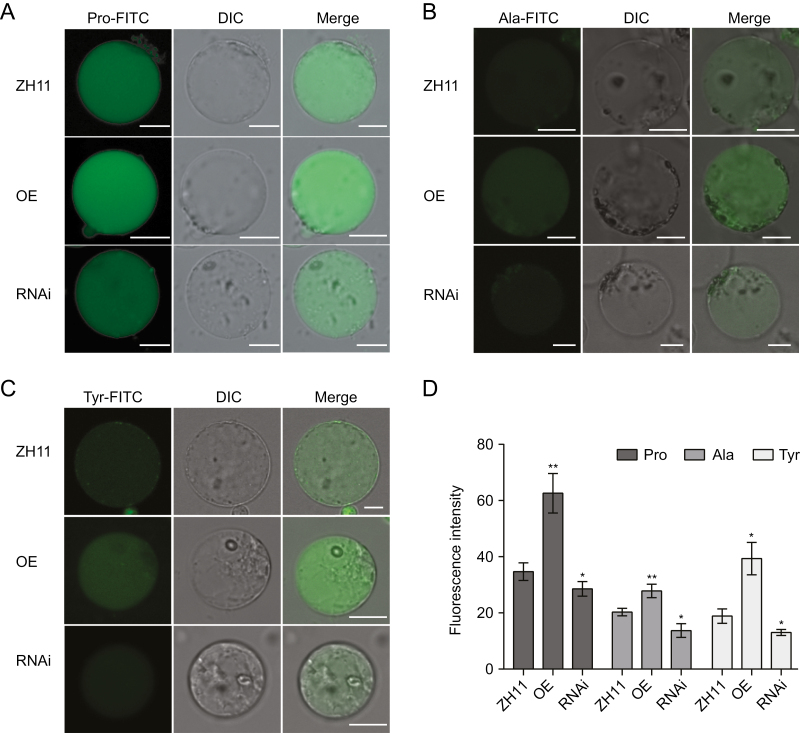
Protoplast amino acid uptake assay in the *OsAAP1* transgenic plants. Fluorescence was detected after culturing protoplasts with FITC-labeled amino acids at 28 °C in the dark for 4 h. Green fluorescence images of the *OsAAP1* OE, RNAi lines and the wild-type ZH11 under Pro–FITC (A), Ala–FITC (B), and Tyr–FITC (C). (D) Statistical analysis of cell fluorescence signal intensity. Fluorescence intensities were normalized to the area of the respective cell by LAX software, and >30 cells were statistically analyzed. Scale bars=5 μm. Values are means ±SD. Differences were analyzed using Student’s *t*-test. ***P<0.001, **P<0.01, *P<0.05.

AtAAP1 mainly transports Pro and Ala in *Saccharomyces cerevisiae* (Lee *et al.*, 2007). To further investigate the function of OsAAP1 in the transportation of neutral amino acids, we performed a protoplast amino acid uptake assay. Rice protoplasts from the OE, RNAi, and wild-type ZH11 lines were cultured with FITC-labeled amino acids, 1 mM Pro-FITC (Fig. 5A), Ala-FITC (Fig. 5B), and Tyr-FITC (Fig. 5C) for 4 h. These results showed that the protoplasts of OE lines had a higher fluorescent signal than that in ZH11 protoplasts, but the fluorescence intensity of the protoplasts from the RNAi lines was weaker than that in the wild-type ZH11 (Fig. 5D), indicating that OsAAP1 may play a role in transporting neutral amino acids (Pro, Ala, and Tyr) into rice plant cells.

### OsAAP1 promoted plant growth by increasing neutral amino acid transportation

To explore the function of OsAAP1 in promoting neutral amino acid transportation, equal amounts (0.5 mM) of Ser, Gly, Ala, Cys, Val, Met, Ile, Leu, Tyr, Phe, and Pro were mixed and exogenously applied to the OE, RNAi, and wild-type ZH11 plants, and their growth phenotypes were evaluated after 3 weeks. The results showed that the growth of the OE lines was notably improved as compared with the wild-type ZH11, but the RNAi lines had much poorer growth than ZH11 (Supplementary Fig. S5A). Analysis of biomass, root number, root length, and plant height also revealed that elevated expression of *OsAAP1* facilitated plant growth by influencing neutral amino acid transportation (Supplementary Fig. S5B–E). Therefore, these results demonstrated that OsAAP1 could transport neutral amino acids and improve rice growth.

**Fig. 6.**
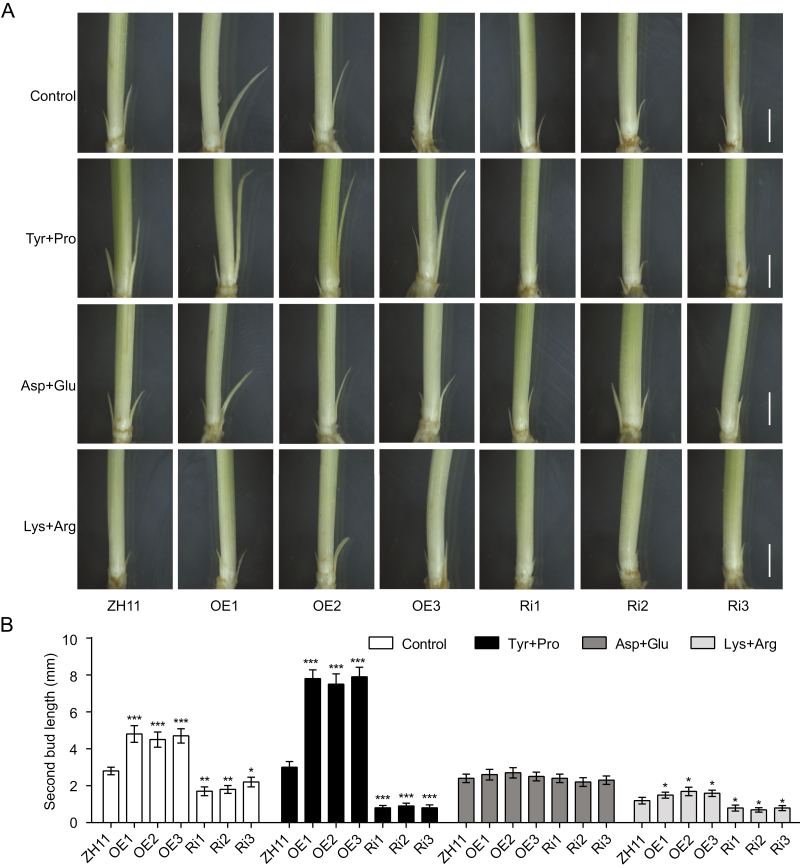
Effect of different types of amino acids on the outgrowth and elongation of the *OsAAP1* transgenic seedlings. (A) The axillary bud phenotypes of the *OsAAP1* OE lines, the RNAi lines, and the wild-type ZH11 seedlings treated with Tyr and Pro, Asp and Glu, or Lys and Arg, respectively. Scale bars represent 3 mm. (B) Second bud length of the rice seedlings in (A). Data are represented as mean ±SD (*n*>15). Differences were analyzed using Student’s *t*-test. ***P<0.001, **P<0.01, *P<0.05.

In addition, we investigated the effect of different amino acid groups on the growth of the *OsAAP1*-related transgenic lines by exogenous application of three groups of amino acids, namely the neutral amino acids Tyr and Pro, the acidic amino acids Asp and Glu, and the basic amino acids Lys and Arg, in the basic nutrient solution. We found that the axillary buds of the *OsAAP1* OE lines were significantly longer than those of the wild-type ZH11 with the neutral amino acid and the basic amino acid groups, while the RNAi lines showed reduced bud outgrowth (Fig. 6A), and quantitative statistical analysis of the second bud length further confirmed the result (Fig. 6B). Moreover, the neutral amino acid group treatment could strongly enhance biomass accumulation in the OE plants but not in the wild-type ZH11, and significantly inhibited the growth and biomass accumulation of the RNAi lines (Supplementary Fig. S6A–D). The basic amino acid treatment had no obvious effect on seedling growth and biomass accumulation of the *OsAAP1* OE lines, but significantly inhibited the growth of the RNAi lines (Supplementary Fig. S6A, B, E, F). The acidic amino acid group treatment had no apparent effect on the rice plant growth, bud outgrowth, and biomass in both the OE lines and the RNAi lines as compared with the wild-type ZH11 (Fig. 6; Supplementary Fig. S6A, B, G, H), indicating that OsAAP1 may not participate in transporting acidic amino acids. Taken together, it was indicated that elevated expression of *OsAAP1* can enhance the transport of the neutral amino acids Tyr and Pro and show a similar phenotype to basic and acidic amino acids compared with the wild-type ZH11 (Supplementary Fig. S7A), leading to biomass accumulation.

As elevated ammonium nitrate could promote plant growth (Li *et al.*, 2016), we tested whether different concentrations (0.25, 1.0, and 4.0 mM) of ammonium nitrate can affect the growth of the wild-type ZH11, and the OE and RNAi lines. We found that the root length of ZH11 varies, with no significant difference under different levels of ammonium nitrate (Supplementary Fig. S7B). The root number of ZH11 was significantly increased under 1.0 mM ammonium nitrate, but not significantly changed under 4.0 mM ammonium nitrate (Supplementary Fig. S7C). The plant heights of ZH11 were increased with both 1.0 mM and 4.0 mM ammonium nitrate (Supplementary Fig. S7D). Furthermore, we observed that the OE lines grew much better than ZH11 under 0.25 mM ammonium nitrate (Supplementary Fig. S8A), whereas the RNAi lines grew worse than the wild type under various N concentrations (Supplementary Fig. S8A–C). Root length (Supplementary Fig. S8D), root number (Supplementary Fig. S8E), and plant height (Supplementary Fig. S8F) were all significantly decreased in the RNAi lines under different concentrations of ammonium nitrate. However, under 0.25 mM ammonium nitrate, they were significantly increased in the OE lines, but no effect was observed with 1.0 mM and 4.0 mM concentrations in the OE lines, except for the root number under 4.0 mM ammonium nitrate.

### Knockout of OsAAP1 significantly reduced tiller number and grain yield

**Fig. 7.**
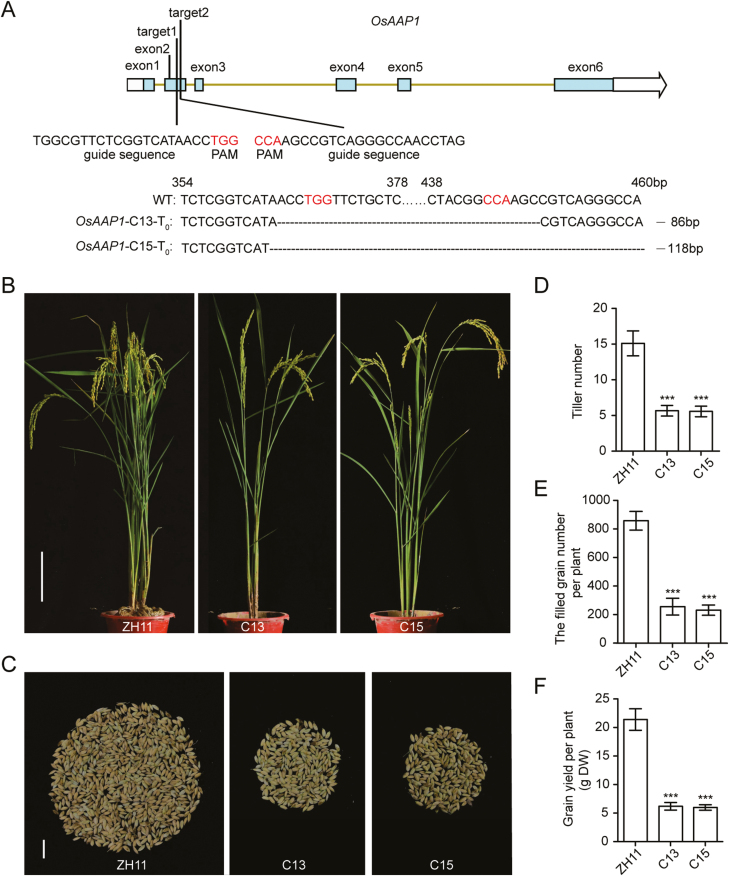
Phenotype of the *OsAAP1*-CRISPR line. (A) Identification of two independent *OsAAP1*-CRISPR lines in rice. ‘–’ indicates the deletion of the nucleotide in the *OsAAP1*-CRISPR T_0_ lines. (B) The phenotype of *OsAAP1*-CRISPR lines. (C) Phenotype of the grain number per plant. (D) Quantification of the tiller number per plant in (B). (E) Quantification of the grain number per plant in (A). (F) Quantification of the grain yield per plant. Scale bars represent 25 cm and 3 cm in (B) and (C), respectively. C13 and C15 indicate two independent *OsAAP1*-CRISPR T_1_ lines. Data are represented as the mean ±SD (*n*>20). Differences were analyzed using Student’s *t*-test. ***P<0.001, **P<0.01, *P<0.05.

Because *OsAAP1* positively regulates tiller number and grain yield per plant, we knocked out *OsAAP1* in the wild-type ZH11 using CRISPR technology to verify the function of this gene in rice. Two target sites were selected for the genomic editing of *OsAAP1* (Fig. 7A). Two independent *OsAAP1* knockout lines, *Osaap1-C13* and *-C15*, with 86 bp and 118 bp deletions, respectively, were identified. *Osaap1-C13* and *-C15* produced decreased tiller number (Fig. 7B, D), filled grain number (Fig. 7C, E), and grain yield per plant at the heading stage (Fig. 7C, F), indicating that knockout of *OsAAP1* can reduce grain yield by inhibiting tiller number, which is consistent with a previous study that showed that *Osaap1* could increase grain yield (Pereira *et al.*, 2015). Thus, these results further support that *OsAAP1* positively regulates tiller number and grain yield in rice.

### Influence of knockout of *OsAAP1* on nitrogen metabolism and plant hormone-related genes in axillary buds

**Fig. 8.**
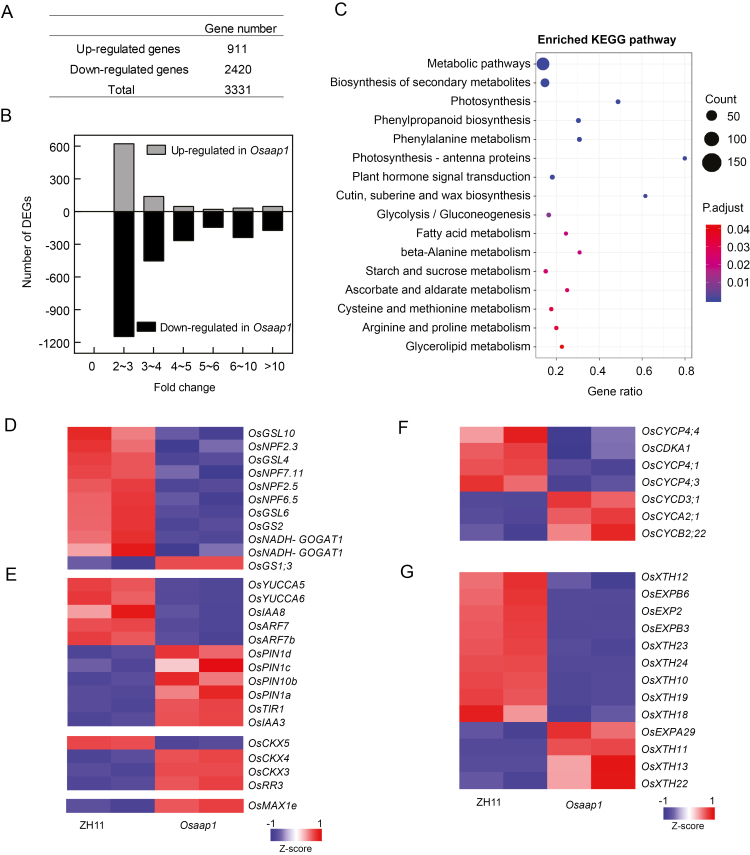
Transcriptome analysis of *Osaap1* in the growing axillary buds. (A) Identification of differentially expressed genes (DEGs) in the axillary buds of *Osaap1* compared with the wild-type ZH11 (adjusted *P*-value <0.05 and fold change >2). (B) Fold change of DEGs in (A). (C) KEGG enrichment analysis of the DEGs in the axillary buds of *Osaap1* compared with the wild-type ZH11. Gene ratio indicates that the ratio of the DEG number and the number of genes has been annotated in this pathway. (D–G) Heatmap visualization of expression profiles of DEGs in nitrogen transport and metabolism (D), auxin, cytokinin, and SL signaling pathways (E), cell expansion (E), and division (F). Red boxes show up-regulation, and green boxes show down-regulation.

To investigate the mechanism of *OsAAP1* in regulating axillary bud outgrowth, we performed RNA-seq using RNA samples from the axillary buds of the *Osaap1* and the wild-type ZH11. A total of 3331 genes were differentially expressed (FDR <0.05 and fold change ≥2) between the two samples, including 911 up-regulated genes and 2420 down-regulated genes (Fig. 8A, B). To understand the biological functions of these DEGs, we performed Kyoto Encyclopedia of Genes and Genomes (KEGG) pathway enrichment analysis. The DEGs were assigned to 21 KEGG pathways, such as metabolic pathways, biosynthesis of secondary metabolites, and plant hormone signal transduction (Fig. 8C), which are similar to the results of the KEGG pathway enrichment analysis of DEGs from the removed panicle compared with *rac*-GR24 treatment (Zha *et al.*, 2019). In addition, we quantified the expression levels of the *OsAAP* family members in the *Osaap1* and the wild-type ZH11, and found that the expression of *OsAAP1* was significantly decreased, whereas there were no significant changes in other *OsAAP* genes (Supplementary Fig. S9).

It is well known that N, acting as an essential nutrient and an environmental factor, can regulate rice tillering. To further investigate the mechanism of the reduced tiller number of *Osaap1*, we analyzed the expression patterns of DEGs in N transport and metabolism, and the heatmap result showed that all of the DEGs had reduced expression, except for *OsGS1;3* (Fig. 8D), which indicated that knockout of *OsAAP1* may affect the expression of other N transport genes and the glutamine synthetases needed for the regulation of the axillary bud outgrowth and achievement of the plant growth requirements for N. In addition, because the phytohormone cytokinin promotes tillering (Dun *et al.*, 2012), while auxin, SL, and ABA can inhibit tillering (Leyser, 2003; Gomez-Roldan *et al.*, 2008; Umehara *et al.*, 2008; Wang *et al.*, 2013; Wang *et al.*, 2018; Hu *et al.*, 2019; Fang *et al.*, 2020), we analyzed related genes in these pathways. The heatmap result showed that *YUCCA* auxin biosynthetic genes were down-regulated in the *Osaap1* plants compared with the wild-type ZH11, indicating that auxin may be accumulated in the axillary buds of *Osaap1*, leading to the up-regulation of the auxin transporter *PIN* genes, and resulting in the inhibition of the axillary bud outgrowth (Fig. 8E). In addition, the increased expression of *OsCKX3*, *OsCKX4*, and the cytokinin signaling negative regulator *OsRR3* may inhibit the cytokinin signaling, leading to the inhibition of axillary bud outgrowth of *Osaap1* (Fig. 8E). Moreover, the expression of the SL biosynthesis gene *OsMAX1e* was increased to inhibit the axillary bud outgrowth of *Osaap1* (Fig. 8E), in agreement with a previous work (Wang *et al.*, 2015). Taken together, *OsAAP1* may promote the axillary bud outgrowth through affecting related signaling pathways that determine tiller number.

## Discussion

In this study, we provide several lines of evidence to support that *OsAAP1* encodes an amino acid transporter to promote tillering and can be potentially used to improve grain yield in rice. First, *OsAAP1* is highly expressed in the roots, axillary buds, leaves, and young panicles, and therefore may participate in rice root, tillering, and seed loading of amino acids. Secondly, OsAAP1 is a membrane protein localized on both the plasma membrane and nuclear membrane to transport amino acids. Thirdly, overexpression of *OsAAP1* improves grain yield by increasing tiller number and filled grain number per plant, whereas both the *OsAAP1* RNAi and knockout lines displayed significantly reduced tiller number and grain yield. In addition, overexpression of *OsAAP1* may promote the transport of neutral amino acids in the straw, especially of Tyr and Pro, to improve seed loading and grain yield, but knockdown of *OsAAP1* enhanced both basic and acidic amino acid levels to balance the amino acids. Finally, *OsAAP1* could significantly improve plant growth under low N conditions.

Overexpression of *OsAAP1* significantly increased the tiller number (Fig. 3A, C), which was consistent with a previous study that suggested that exogenously applied Pro could improve the shoot regeneration frequency of rice *in vitro* (Pawar *et al.*, 2015) and supports that N nutrition promotes shoot branching in rice. In Arabidopsis, *Ataap1* or *Ataap8* mutants had decreased contents of neutral amino acids in the embryo and abnormal seed development (Schmidt *et al.*, 2007; Sanders *et al.*, 2009). We found that the *OsAAP1* RNAi lines had decreased neutral amino acid content in the straw or seed, especially of Pro and Tyr, and a reduced response to the basic amino acids Lys and Arg, which may lead to the inhibited plant growth. (Fig. 4C, D). It was reported that knockdown or knockout of *OsAAP3* or *OsAAP5*, whose products mainly transport basic amino acids (Lys and Arg), could promote axillary bud outgrowth and enhance grain yield and N use efficiency (Taylor *et al.*, 2015; Lu *et al.*, 2018; Wang *et al.*, 2019). The reduced content of neutral amino acids and the accumulation of basic amino acids in the knockdown line of *OsAAP1* may explain the reduced growth of the RNAi lines (Figs 3A, 4C, D).

The reduced tiller number in *OsAAP1* RNAi lines and CRISPR lines may be caused by reducing axillary bud outgrowth. It has been reported that N deficiency limits axillary bud outgrowth by suppressing cell division-determined elongation (Luo *et al.*, 2017). Furthermore, the P-type cyclins were found to interact with cyclin-dependent kinase A-1 (CDKA;1) and may regulate cell division based on nutrient status (Acosta *et al.*, 2004, Deng *et al.*, 2014). Thus, we analyzed the cell cycle-related genes in *Osaap1*; interestingly, the results showed that *Cyclin-P4-1* (*OsCYCP4;1*), *OsCYCP4;3*, *CYCP4;4*, and *OsCDKA1* were all down-regulated in *Osaap1* compared with the wild type (Fig. 8F). In addition, N deficiency influenced cell wall modifications, including xyloglucan endotransglucosylase/hydrolase (XTH) and expansins (EXPs), resulting in the inhibition of cell enlargement and expansion by the decreased biosynthesis of xylogucans, and reduced plant cell wall extensibility (Qin *et al.*, 2019). Therefore, we examined the expression of *OsXTH* and *OsEXP* genes in the DEGs of *Osaap1* compared with those in the wild-type ZH11, and found that most of them were down-regulated (Fig. 8G), which was in accordance with previous work. Finally, *OsAAP1* may influence the N transport and metabolism genes, and the auxin, cytokinin, and SL signaling-related genes in the axillary bud of rice. However, it is still an open question as to the molecular mechanism of how *OsAAP1* regulates rice tillering, thus requiring further investigation.

## Supplementary data

Supplementary data are available at *JXB* online.

Fig. S1. The root of ZH11 showed no GUS signal compared with the *pOsAAP1::GUS* lines.

Fig. S2. Relative *OsAAP1* expression in different tissues and with different phytohormone treatments.

Fig. S3. Relative *OsAAP1* expression in the OE and RNAi lines.

Fig. S4. Total nitrogen content and NUtE of the *OsAAP1* transgenic plants.

Fig. S5. Exogenously applied neutral amino acids promote the growth of *OsAAP1* OE rice plants.

Fig. S6. The *OsAAP1* transgenic plants respond to different types of amino acids.

Fig. S7. The wild-type ZH11 response to different N source and N content conditions.

Fig. S8. Effect of different concentration of NH_4_NO_3_ on the growth of the *OsAAP1* transgenic seedlings.

Fig. S9. Fold change of the *OsAAP* gene family in the axillary buds of *Osaap1* compared with the wild-type ZH11.

Table S1. Amino acid concentrations in the straw (mg g DW^–1^).

Table S2. Amino acid concentrations in the grain (mg g DW^–1^).

eraa256_suppl_Supplementary_Material

## Data Availability

The raw data collected from RNA-seq are available at the National Center for Biotechnology Information (NCBI): https://dataview.ncbi.nlm.nih.gov/object/PRJNA623117?reviewer=nljkvvj6sgfi8jv27doqab0sfa.

## Abbreviations:

AAP: amino acid permease
AAT: amino acid transporter protein
ABA: abscisic acid
ABRE: ABA-responsive element
ACE: ACGT-containing element
CRISPR: clustered regularly interspaced short palindromic repeat
FITC: fluorescein isothiocyanate
GFP: green fluorescent protein
GUS: β-glucuronidase
NPF: nitrate transporter 1/peptide transporter family
NUtE: nitrogen utilization efficiency
OE: overexpression
qRT-PCR: quantitative real-time PCR
SL: strigolactone
