# Supplementary material for: The amino acid transporter AAP1 mediates growth and grain yield by regulating neutral amino acid uptake and reallocation in Oryza sativa
Source: J Exp Bot. 2020 Jun 2;71(16):4763–77. doi: 10.1093/jxb/eraa256 (PMC7410190; doi:10.1093/jxb/eraa256)
Supplement: eraa256_suppl_Supplementary_Material [file eraa256_suppl_supplementary_material.pdf]

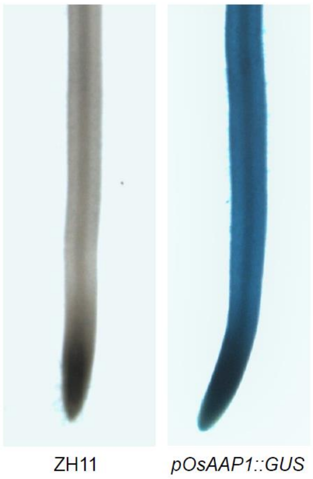

Fig. S1 The root of ZH11 showed no GUS signal compare with the *pOsAAP1::GUS* lines.

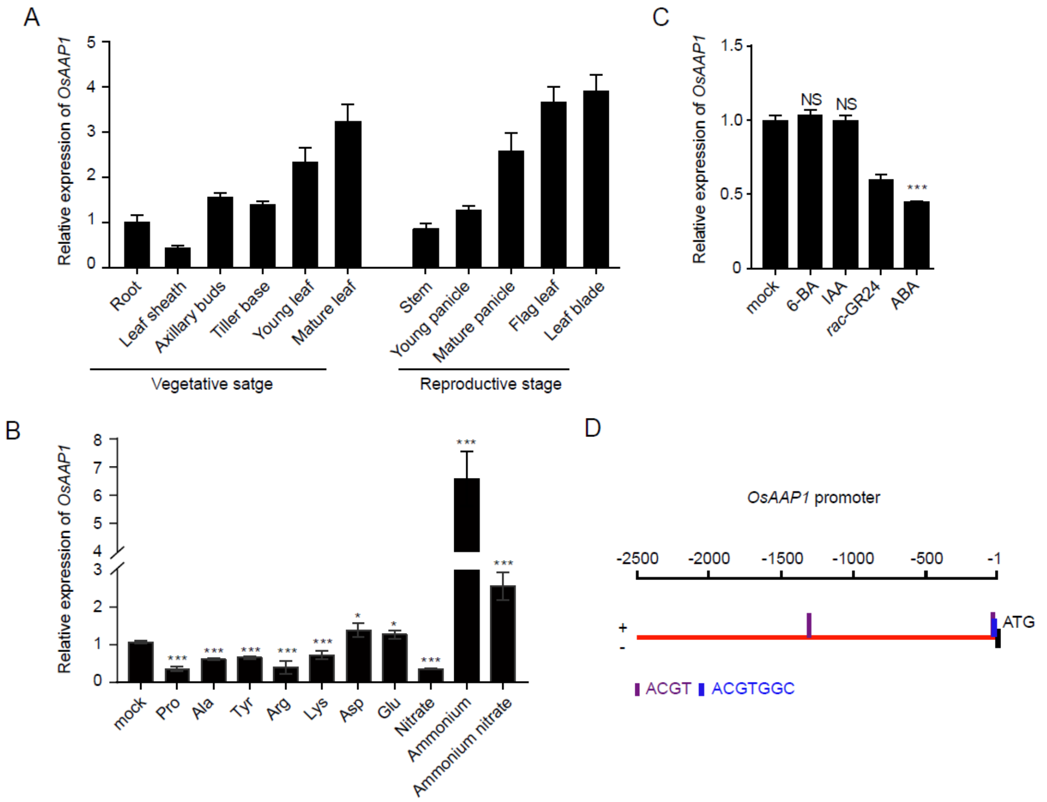

Fig. S2 Relative *OsAAP1* expression in different tissues and with different phytohormone treatments. (A) Relative *OsAAP1* expression in diverse tissues at different vegetative and reproductive stages in the ZH11 rice. (B) Relative *OsAAP1* expression of the ZH11 rice seedlings treated with various nitrogen resources. The 3-week-old ZH11 rice seedlings were cultured in basic nutrient solution with 1 mM NH_4NO_3, then transferred to N-free basic nutrient solution for 3 days. The N-starved rice plants were transferred to N-free basic nutrient solution with or without 2 mM Pro, Ala, Tyr, Arg, Lys, Asp, Glu, $NaNO_3$, 1 mM $(NH_4)_2SO_4$, and 1 mM NH_4NO_3 for 4 h, respectively. The roots were sampled for the RNA extraction and qPCR assay. (C) Relative *OsAAP1* expression of the ZH11 rice seedlings treated with mock, 10 µM 6-BA, 10 µM IAA, 1 µM GR24, 1 µM ABA for two hours. (D) Analysis of the ABRE sequences in the promoter of *OsAAP1*. The purple bar represents ABRE core sequence (ACGT) and ABRE element (ACGTGGC). Data are represented as mean ± SD (n=3). Differences were analyzed using Student's t-test. *** $P< 0.001$, $^{**}P < 0.01$, NS, Non-significant, $P > 0.05$.

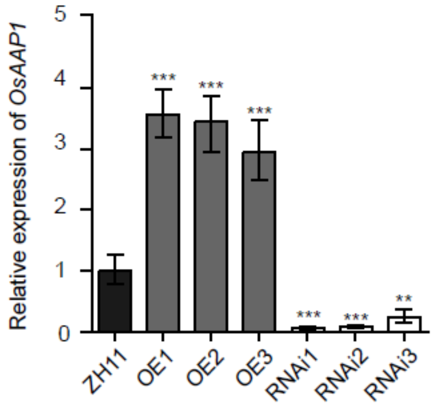

Fig. S3 Relative *OsAAP1* expression in the OE and RNAi lines. OE1-OE3 indicate the different *OsAAP1*-overexpression lines, and RNAi1-RNAi3 indicate the different OsAAP1-RNAi lines. ZH11 indicates the wild-type. Data are represented as mean ± SD (n=3). Differences were analyzed using Student's t-test. *** P < 0.001, ** P < 0.01.

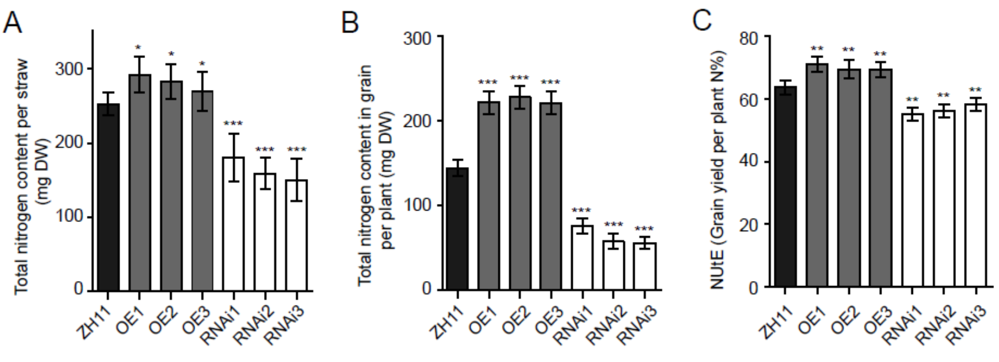

Fig. S4 Total nitrogen content and NUE of the *OsAAP1* transgenic plants. (A) Total nitrogen content per straw (mg DW). (B) Total nitrogen content in grain per plant (mg DW) of the *OsAAP1* transgenic plants. (C) NUtE (Nitrogen utilization efficiency) of the grain yield per plant of the *OsAAP1* transgenic plants. Data are represented as mean ± SD (n>20). Differences were analyzed using Student's t-test. $^{***}P < 0.001$, $^{**}P < 0.01$, $^{*}P < 0.05$.

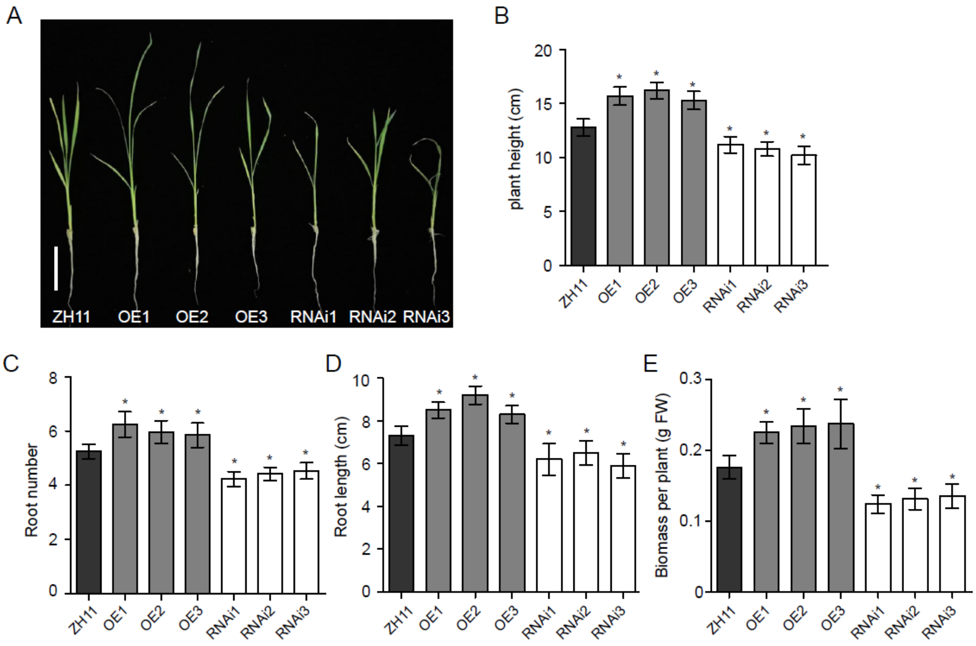

Fig. S5 Exogenously applied neutral amino acids promote the growth of *OsAAP1* OE rice plants. (A) the phenotype of the *OsAAP1* OE lines, the RNAi lines, and the wild type ZH11 treated with 0.5 mM neutral amino acids (Mixed with equal amount of Ser, Gly, Ala, Cys, Val, Met, Ile, Leu, Tyr, Phe and Pro). Scale bar represents 5 cm. (B-E) biomass, root number, root length and plant height of plants in (A). Data are represented as mean ± SD (n>20). Differences were analyzed using Student's t-test. *** $P < 0.001$, ** $P < 0.01$, * $P < 0.05$.

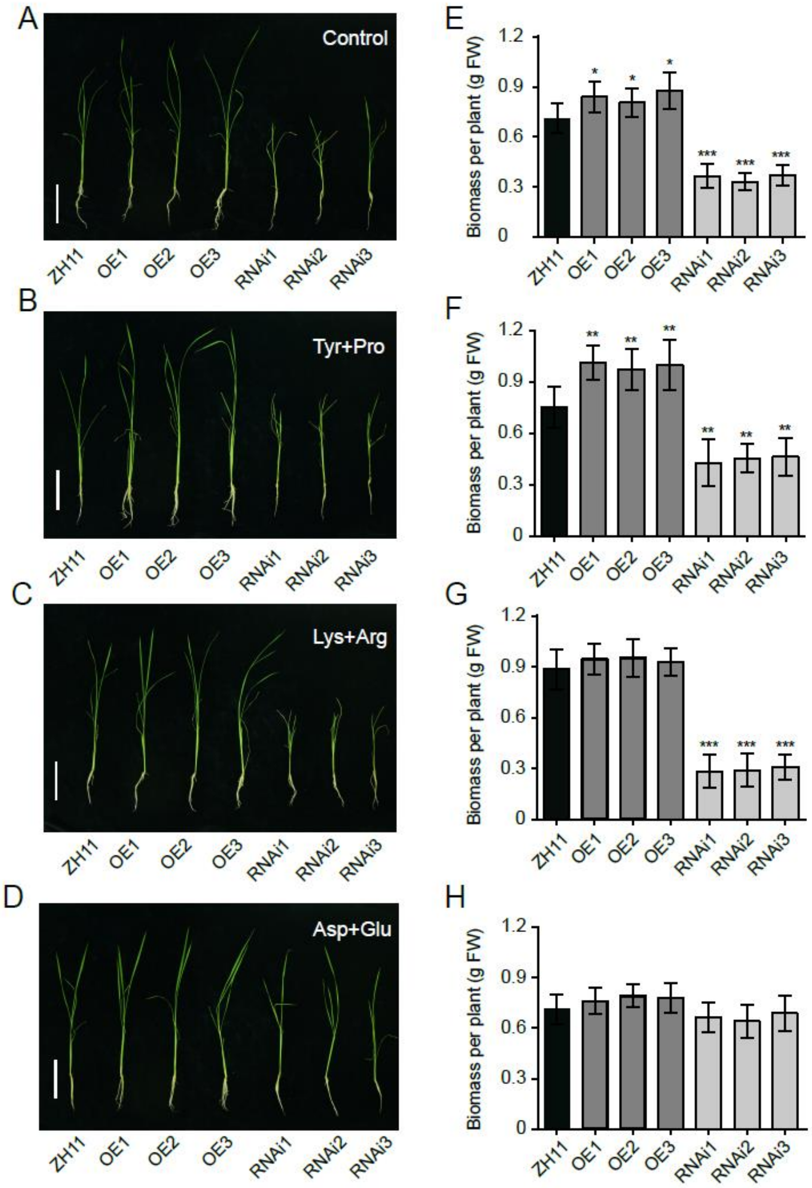

Fig. S6 The *OsAAP1* transgenic plants respond to different types of amino acids. (A) The phenotype of the *OsAAP1* OE lines, the RNAi lines, and the wild type ZH11. (B) The phenotype of the *OsAAP1* OE lines, the RNAi lines, and the wild type ZH11 treated with Tyr and Pro. (C) The phenotype of the *OsAAP1* OE lines, the RNAi lines, and the wild type ZH11 treated with Lys and Arg. (D) The phenotype of the *OsAAP1* transgenic plants treated with Asp and Glu. (E-H) Biomass per plant corresponding to the treatments on the left panels of (A-D), respectively. Scale bars represent 10 cm in (A-D). Data are represented as mean ± SD (n>20). Differences were analyzed using Student's t-test. *** $P < 0.001$, ** $P < 0.01$, * $P < 0.05$.

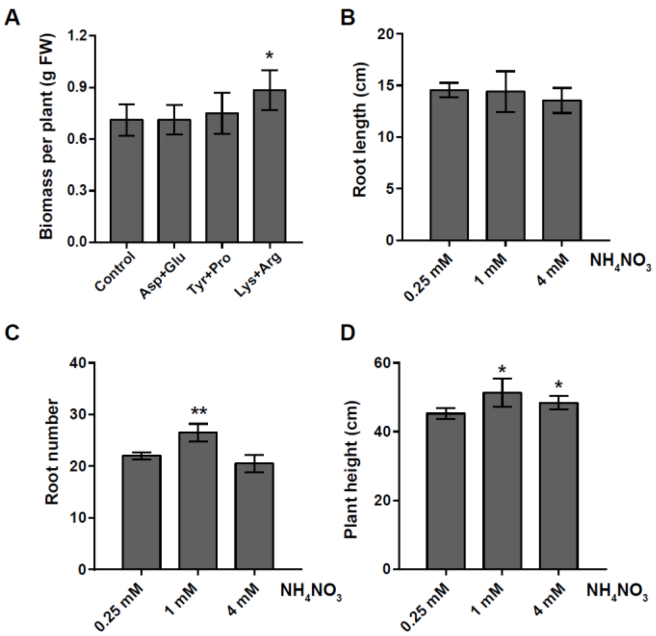

Fig. S7 the wild type ZH11 response to different N source and N content conditions. (A) Biomass per plant of ZH11 under different groups of amino acid. (B-D) Root length, root number and plant height of ZH11 under elevated content of NH_4NO_3. Data are represented as mean ± SD (n>20). Differences were analyzed using Student's t-test. ** $P < 0.01$, * $P < 0.05$.

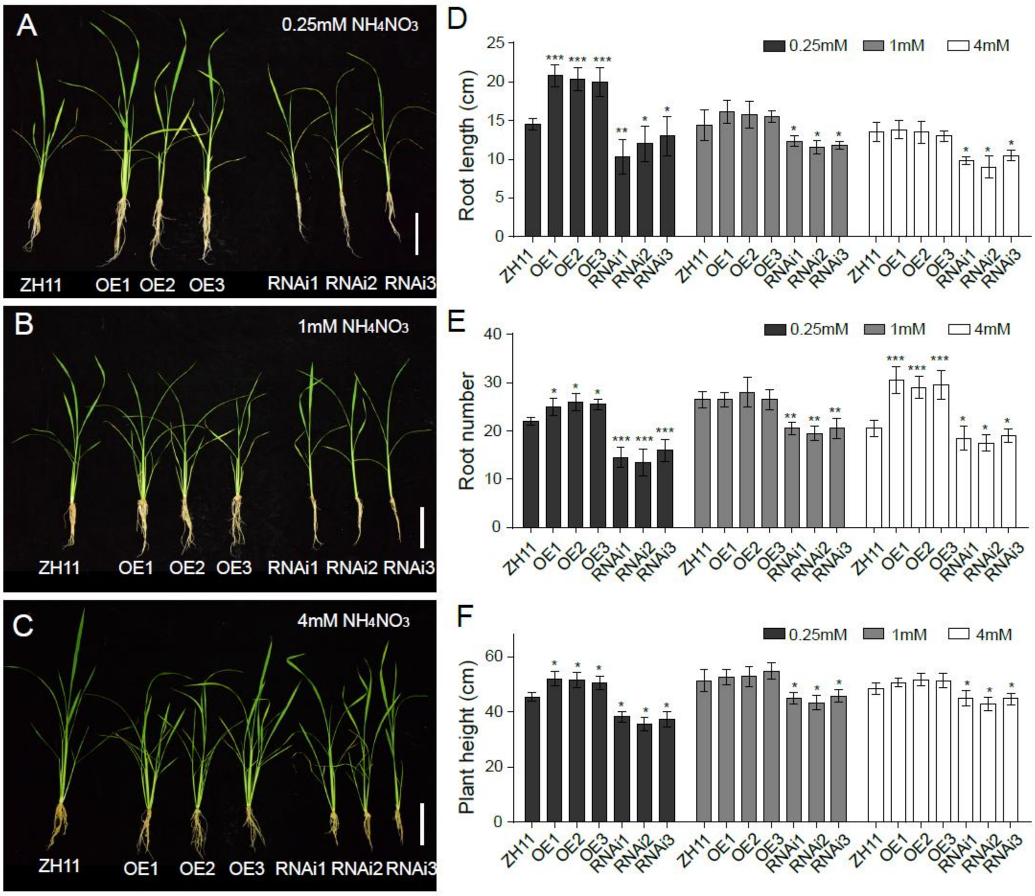

Fig. S8 Effect of different concentrations of NH_4NO_3 on the growth of the *OsAAP1* transgenic seedlings. (A-C) The phenotypes of the *OsAAP1* transgenic seedlings under 0.25 mM, 1 mM and 4 mM NH_4NO_3. Scale bars represent 5 cm. (D-F) Root length, root number and plant height of the *OsAAP1* transgenic seedlings in (A) (B), or (C), respectively. Data are represented as mean ± SD (n>20). Differences were analyzed using Student's t-test. *** $P < 0.001$, ** $P < 0.01$, * $P < 0.05$.

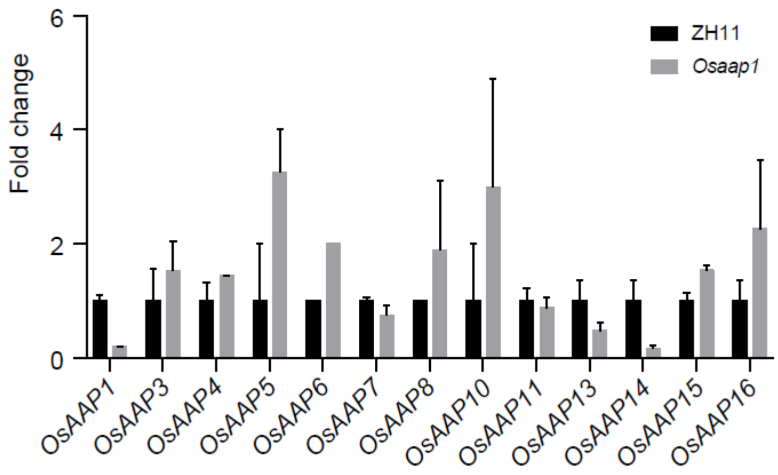

Fig. S9 Fold change of the OsAAPs family in the buds of *Osaap1* compared with the wild type ZH11. The expression *OsAAP2*, *OsAAP9*, *OsAAP12*, *OsAAP17*, *OsAAP18*, and *OsAAP19* were not detected both in the buds of *Osaap1* and the wild type ZH11. Data are represented as mean ± SEM.

Table S1 Amino acid concentrations in the straw (mg/g DW)

	WT		OE1		OE2		RNAi1		RNAi2	
	Mean	SD	Mean	SD	Mean	SD	Mean	SD	Mean	SD
Asp	3.29	0.08	3.51	0.46	3.50	0.22	6.98	0.97	6.17	0.55
Thr	7.18	0.21	7.38	0.47	7.36	0.46	8.31	0.84	8.58	0.94
Ser	6.82	0.40	6.97	0.45	6.82	0.27	5.47	0.51	5.13	0.53
Glu	4.02	0.28	3.74	0.56	3.52	0.39	5.49	0.79	5.31	0.21
Gly	7.26	0.58	7.36	0.72	7.06	0.78	4.40	0.24	5.25	0.96
Ala	13.46	1.03	13.60	1.08	13.79	1.25	10.05	0.62	12.18	0.44
Cys	3.10	0.43	3.16	0.34	3.27	0.44	1.71	0.72	2.16	0.23
Val	10.22	0.53	10.39	0.82	10.13	0.83	5.46	1.07	6.94	1.09
Met	5.78	0.22	5.85	0.70	5.65	0.56	1.58	0.30	1.68	0.29
Ile	1.10	0.04	0.95	0.14	0.91	0.09	0.75	0.11	0.45	0.08
Leu	2.93	0.26	2.85	0.25	2.96	0.25	2.01	0.44	2.15	0.22
Tyr	1.65	0.17	2.46	0.35	2.50	0.21	1.10	0.35	1.32	0.18
Phe	2.88	0.24	2.75	0.26	2.79	0.27	2.32	0.23	2.11	0.44
Lys	1.18	0.18	0.96	0.13	0.90	0.17	2.23	0.39	1.91	0.17
His	0.82	0.17	0.62	0.07	0.42	0.03	0.97	0.16	0.95	0.15
Arg	2.95	0.28	2.18	0.26	2.19	0.35	12.42	1.67	10.99	1.60
Pro	4.67	0.41	13.27	0.80	12.29	0.70	2.22	0.39	2.78	0.51

Table S2 Amino acid concentrations in the grain (mg/g DW)

	WT		OE1		OE2		RNAi1		RNAi2	
	Mean	SD	Mean	SD	Mean	SD	Mean	SD	Mean	SD
Asp	6.30	0.49	6.27	0.74	6.51	0.78	7.22	1.35	7.63	0.70
Thr	3.00	0.24	2.97	0.44	3.04	0.33	3.45	0.37	3.13	0.48
Ser	3.67	0.31	3.68	0.58	3.71	0.42	3.31	0.45	3.17	0.45
Glu	9.77	0.60	9.83	0.79	9.21	0.75	11.50	0.92	10.63	0.85
Gly	9.78	0.74	9.56	0.60	9.82	0.64	8.05	0.87	8.44	0.73
Ala	4.59	0.37	4.54	0.41	4.68	0.63	4.17	0.68	4.12	0.47
Cys	0.78	0.13	0.77	0.34	0.65	0.22	0.55	0.25	0.59	0.29
Val	4.82	0.39	4.88	0.60	5.07	0.88	4.78	0.70	4.65	0.88
Met	1.01	0.15	0.92	0.11	1.07	0.28	1.11	0.12	0.93	0.12
Ile	1.49	0.13	1.44	0.22	1.56	0.30	1.39	0.35	1.30	0.42
Leu	5.85	0.30	5.77	0.75	5.84	0.66	2.38	0.75	2.85	0.73
Tyr	2.87	0.31	2.83	0.43	2.95	0.45	2.21	0.51	2.16	0.45
Phe	3.55	0.32	3.58	0.54	3.73	0.42	2.15	0.69	2.40	0.46
Lys	2.57	0.22	2.50	0.43	2.37	0.31	2.93	0.32	2.95	0.38
His	1.82	0.11	1.84	0.12	1.89	0.28	1.48	0.55	1.54	0.37
Arg	5.81	0.52	5.83	0.68	6.09	0.74	6.57	0.73	6.72	0.63
Pro	2.24	0.24	2.74	0.29	2.83	0.25	1.86	0.29	1.89	0.23
